# Aging-Associated Cognitive Decline is Reversed by D-Serine Supplementation

**DOI:** 10.1523/ENEURO.0176-22.2022

**Published:** 2022-06-03

**Authors:** L. Nava-Gómez, I. Calero-Vargas, F. Higinio-Rodríguez, B. Vázquez-Prieto, R. Olivares-Moreno, J. Ortiz-Retana, P. Aranda, N. Hernández-Chan, G. Rojas-Piloni, S. Alcauter, M. López-Hidalgo

**Affiliations:** 1 Escuela Nacional de Estudios Superiores, Universidad Nacional Autónoma de México, Querétaro 76230, México; 2 Facultad de Medicina, Universidad Autónoma de Querétaro, Querétaro 76126, México; 3 Instituto de Neurobiología, Universidad Nacional Autónoma de México, Querétaro 76230, México; 4 Facultad de Ciencias Naturales, Universidad Autónoma de Querétaro, Querétaro 76230, México; 5 Facultad de Ingeniería, Universidad Autónoma de Querétaro, Querétaro 76010, México

**Keywords:** aging, cognitive flexibility, D-serine, fMRI, functional brain connectivity

## Abstract

Brain aging is a natural process that involves structural and functional changes that lead to cognitive decline, even in healthy subjects. This detriment has been associated with NMDA receptor (NMDAR) hypofunction because of a reduction in the brain levels of D-serine, the endogenous NMDAR co-agonist. However, it is not clear whether D-serine supplementation could be used as an intervention to reduce or reverse age-related brain alterations. In the present work, we aimed to analyze the D-serine effect on aging-associated alterations in cellular and large-scale brain systems that could support cognitive flexibility in rats. We found that D-serine supplementation reverts the age-related decline in cognitive flexibility, frontal dendritic spine density, and partially restored large-scale functional connectivity without inducing nephrotoxicity; instead, D-serine restored the thickness of the renal epithelial cells that were affected by age. Our results suggest that D-serine could be used as a therapeutic target to reverse age-related brain alterations.

## Significance Statement

Age-related behavioral changes in cognitive performance occur as a physiological process of aging. Then, it is important to explore possible therapeutics to decrease, retard or reverse aging effects on the brain. NMDA receptor (NMDAR) hypofunction contributes to the aging-associated cognitive decline. In the aged brain, there is a reduction in the brain levels of the NMDAR co-agonist, D-serine. However, it is unclear whether chronic D-serine supplementation could revert the age-detriment in brain functions. Our results show that D-serine supplementation reverts the age-associated decrease in cognitive flexibility, functional brain connectivity, and neuronal morphology. Our findings raise the possibility that restoring the brain levels of D-serine could be used as a therapeutic target to recover brain alterations associated with aging.

## Introduction

Human life expectancy has increased dramatically in the last decades (Bloom and Luca, 2016), although healthy life expectancy has not (Jager and Fraser, 2017). As the rest of the body, the brain also ages affecting multiple domains, such as sensory perception, motor coordination, learning and memory performance, and executive functions like attention and cognitive flexibility (Casjens et al., 2018; Lacreuse et al., 2018; P. Wu et al., 2020; Cai et al., 2022). Aging-associated cognitive decline is accompanied by alterations in the complexity of neuron morphology, including dendritic arborization and spine density, which is instrumental for proper neural network function.

Although aging is a multifactorial process, several lines of evidence indicate that a hypofunction of NMDA receptors (NMDARs) contributes to age-related cognitive decline (Clayton et al., 2002; Foster, 2007; Mostany et al., 2013; Kumar and Foster, 2019). NMDARs are critical in regulating activity-dependent synaptic plasticity and are involved in many cognitive functions (Kuehl-Kovarik et al., 2000; Paoletti and Neyton, 2007; Forsyth et al., 2015; Bye and McDonald, 2019; Banks and Bashir, 2021). In addition to glutamate, NMDAR activation requires the binding of a co-agonist: glycine or D-serine (Schell et al., 1995; Bergeron et al., 1998; Pollegioni and Sacchi, 2010; Cummings and Popescu, 2015; Guo et al., 2017; Bodner et al., 2020). However, in the aged brain, D-serine (but not glycine) concentration and content is reduced (Junjaud et al., 2006; Mothet et al., 2006; Potier et al., 2010), resulting in a decrease of NMDAR-dependent synaptic plasticity (Junjaud et al., 2006; Potier et al., 2010; Turpin et al., 2011; Ploux et al., 2021), dendrite complexity and cognitive impairment (Rowland et al., 2005; Lin et al., 2014). D-serine supplementation is essential for the induction of long-term potentiation and prevents oxidative stress-related deficits of synaptic plasticity in hippocampal slices of young animals (Henneberger et al., 2010; Potier et al., 2010; Haxaire et al., 2012; Orzylowski et al., 2021). Furthermore, D-serine treatment in patients with schizophrenia has been successful in improving cognitive functions that are characterized by NMDAR hypofunction (Coyle, 1996; Labrie et al., 2012; Cho et al., 2016). Aside from this evidence, it is still unclear whether the cognitive decline in aging is associated with decreased availability of D-serine and whether chronic D-serine supplementation could revert the age-related decline in cognitive flexibility in senescent rats, and if so, how it affects neuronal morphology and brain functional connectivity. Here, we showed that chronic D-serine supplementation restores the cognitive flexibility, frontal neuronal spine density, and large-scale functional connectivity that is affected by aging.

## Materials and Methods

### Subjects

All experimental procedures were performed in accordance with the NIH *Guide for the Care and Use of Laboratory Animals* and were approved by the Instituto de Neurobiología at Universidad Nacional Autónoma de México (No. 043). Experiments were performed in young (six to eight months old, *n* = 36), middle-aged (18–20 months old, *n* = 49), and aged (24–26 months old, *n* = 33) male Wistar rats (350–400 g). Rats were paired-housed in a temperature-controlled vivarium under a 12/12 h light/dark cycle (lights on at 7 A.M.) and were food restricted to ∼85% of their basal (350–400 g) body with free access to water.

### D-serine supplementation

All rats were randomly assigned into either, control (receiving vehicle) or treatment (receiving D-serine, Sigma-Aldrich, S4250) groups. D-serine was dissolved in the drinking water on a daily basis. The weight and water consumption were monitored per rat and the amount of D-serine was adjusted accordingly to provide a daily supplementation of a dose of 300 mg/kg of body weight or 30 mg where indicated. We did not observe any change in the water consumption because of D-serine supplementation.

### Apparatus

Classical conditioning operant chambers were used to evaluate behavior in a sound-attenuating enclosure. Chambers were constructed with Plexiglas walls and ceiling and with metal grid flooring (29 cm long, 24 cm wide, 29 cm high). The front wall was equipped with retractile response levers at the left and right sides, both with one 5V white LED overhead. A feeder delivered one food pellet per correct answer in a compartment located between the two levers. All chambers were controlled with an Arduino microcontroller board and Visual Basic homemade applications.

### Reversal learning task

#### Training

**Figure 1.**
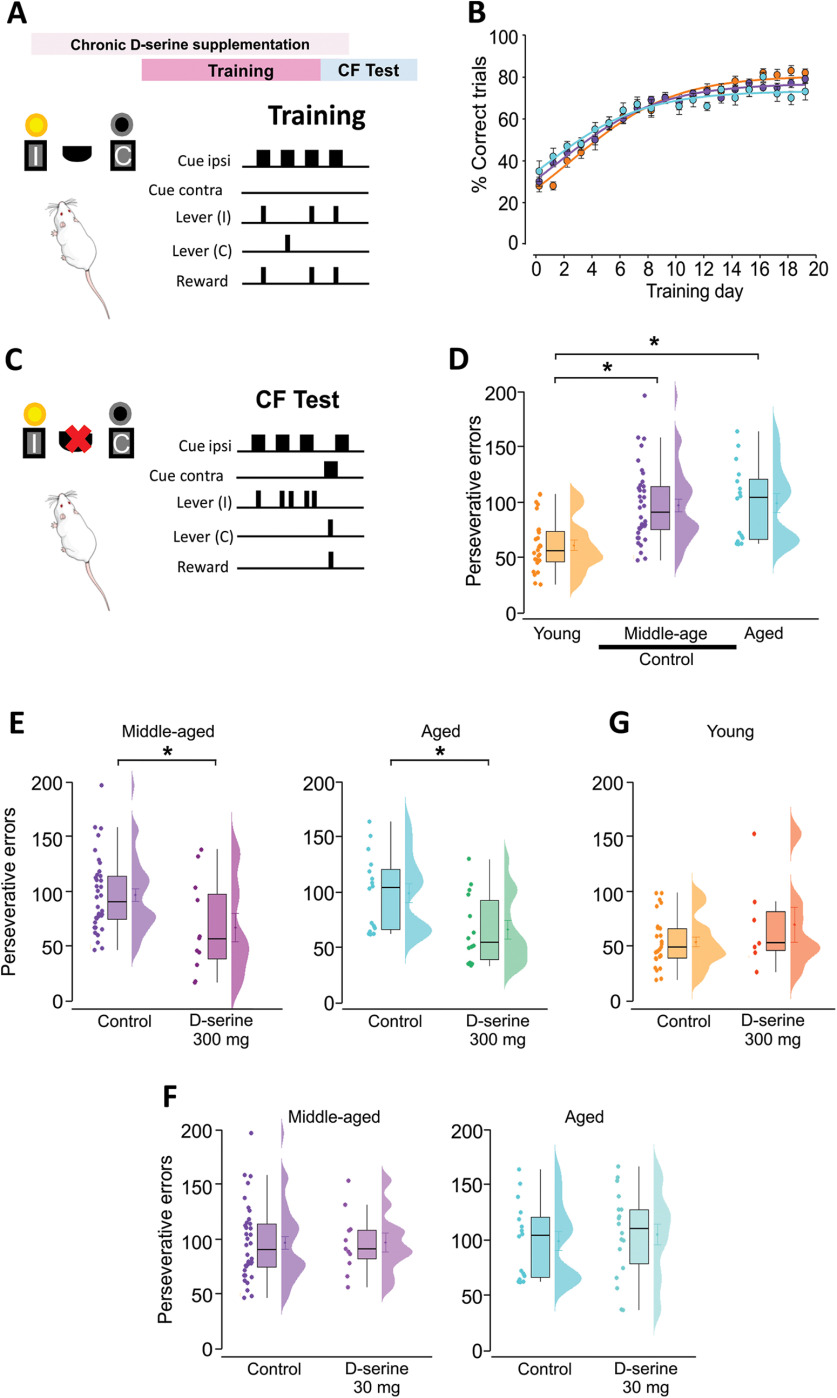
Aging-associated cognitive flexibility decline is prevented by D-serine supplementation. ***A***, Behavioral task design during training sessions where the reward is delivered by pressing the lever ipsilateral (I) to the light. ***B***, time course of correct trials during the training sessions. ***C***, During the cognitive flexibility (CF) test the reward is delivered after pressing the lever contralateral to the light. ***D***, Middle-aged and aged rats showed a significant increase in the number of perseverative errors compared with young rats. ***E***, Middle-aged and aged rats supplemented with D-serine (300 mg/kg of weight) had significantly less perseverative errors during the evaluation of cognitive flexibility in comparison to those receiving vehicles. ***G***, Young rats receiving D-serine (300 mg/kg of weight) did not show significant differences when compared with young control rats. ***F***, Middle-aged and aged rats supplemented with a lower dose of D-serine (30 mg/kg of weight) had no differences in the perseverative errors during the evaluation of cognitive flexibility in comparison to those receiving vehicles. One-way ANOVA for multiple comparisons. Two-tailed *t* test for comparison between two groups; *p ≤ 0.05.

All rats were manipulated and habituated to the experimenter a month before starting the training. Two days before the experiments, the rats were moved to a vivarium next to the experimentation room. The rats were trained in two sequential phases; during phase 1 (one to five sessions), they were exposed to the chamber with both lights on and the levers extended. The rats were conditioned in a 1:1 fixed-ratio schedule of reinforcements where pressing any lever resulted in the delivery of one pellet onto the plate. The counts for each lever press were recorded and the session ended either after 30 min or when the rat pressed any lever 50 times (50 reinforcers). During this phase, we identified the preferred lever (i.e., the one pressed at least 70% of the time). This phase ends when rats reached 50 reinforcers for two consecutive days. In phase 2 (20 sessions), both levers were extended but only the preferred lever pressed in response to the ipsilateral light (10 s) was reinforced with food delivery (Fig. 1*A*). Pressing the preferred lever with the light off or pressing the contralateral lever resulted in no pellet delivery and the retraction of the lever; this was counted as an error. Following a lever press, the levers were retracted for a 2-s time-out period. The sessions ended after 30 min or when the rat pressed any lever 120 times. The rats reached the criterion level when they achieved at least 70% of correct trials in three consecutive days.

#### Cognitive flexibility test

Once the criterion level of performance was achieved, the response outcome was reversed and the rats no longer received a food pellet after pressing the ipsilateral lever. Instead, the rats received a pellet after pressing the contralateral lever (Fig. 1*C*). If the rat persisted in responding to the previously reinforced stimulus (pressing the ipsilateral lever) after 10 min of starting the session, the perseverative errors were counted. Perseverative errors were counted as a negative relation with cognitive flexibility; the more perseverative errors the less cognitive flexibility.

### Attention test

After the rats were evaluated in the reversal learning task, they were retrained to press the lever ipsilateral to the light for one session. During this session, the rats again reached 70% of correct trials. To evaluate the attention components (correct trials and reaction time of the response), the lights were randomly presented either to the left or right side for 0.5 s. Once the light was turned off, both levers were extended and the rat had to select the lever ipsilateral to the light (by pressing it) to receive a pellet. This was counted as a correct trial. The reaction time of the response was counted as the amount of time the rat pressed the lever once the light was turned off.

### Resting-state fMRI acquisition

Resting-state fMRI uses blood oxygenation level-dependent (BOLD) signal correlations as a measure of functional brain connectivity (Biswal et al., 1995; Gorges et al., 2017). We used a T2-weighted magnetic resonance imaging sequence acquired with a 7 Tesla magnetic resonance scanner (Bruker BioSpin Pharmascan 70/16US). Subsequently, functional connectivity between a set of brain regions known to be related to cognitive functions (see below), such as cognitive flexibility and a high expression of NMDARs, was performed to characterize their age-related changes and the effects of D-serine on the aged rat brain.

The rats were food-deprived for a minimum of 12 h before starting the procedures. Anesthesia was induced with isoflurane (5%; Sofloran; PiSA) enriched with oxygen for 5 min. Once the animals were unresponsive, dexmedetomidine was administered (subcutaneous; Dexdomitor; Zoetis, 0.007 mg/kg) and the rats were placed in the scanner with the head fixed and maintained with isoflurane (0.25–0.50%) during the scanning session. Heart rate, breath rate, and spO2 were monitored continuously to assess the depth of anesthesia and general physiological condition of the animals. Body temperature was maintained by circulating warm water within the animal holder.

#### MRI scan parameters

Paravision-6 software (Bruker) was used in this project. A 2 × 2 array surface coil was positioned on the rat’s head, in combination with a 70 mm transmission/reception coil to acquire anatomic and functional imaging. An anatomic scan was first acquired using a spin-echo rapid acquisition with refocused echoes (Turbo-RARE) sequence with the following parameters: repetition time (TR) = 4213 ms, echo time (TE) = 33 ms, RARE factor = 16, number of averages (NA) = 2, field of view (FOV) = 30 × 30 mm^2^, matrix dimension (MD) = 144 × 160, slice thickness = 1 mm, resulting in 2D isotropic voxels of 0.117 × 0.117 mm. Local field homogeneity was optimized within an ellipsoid covering the skull using previously acquired field maps before the fMRI sequence. BOLD rsfMRI was acquired using a 10-min free induction decay echo-planar imaging (FID-EPI) sequence: read orientation left-right, gap 0.200 mm, TR = 1000 ms, TE = 20 ms, flip angle (FA) = 60°, FOV = 30 × 30 mm^2^, in-plane resolution of 0.469 × 0.469 mm, and slice thickness of 1 mm.

#### Preprocessing

Data preprocessing was performed using FSL v5.0.9. library. The first five volumes of each functional series were discarded. Datasets underwent slice-timing correction and motion correction taking the first nondiscarded volume as reference. This reference volume was also used to determine the rigid-body transformation to the corresponding anatomic image. This transformation was combined with an affine transformation from the anatomic image to the Tohoku University rat brain atlas. To minimize the effect of physiological noise, we regressed out the first five eigenvectors (time series) within a mask of nongray brain regions (Behzadi et al., 2007), since recent findings have shown that regressing out vascular, ventricle, and white matter signal enhances functional connectivity specificity in rodent datasets (Grandjean et al., 2020). The resulting datasets were bandpass filtered to retain frequencies between 0.01 and 0.1 Hz (Gorges et al., 2017). Finally, smoothing was applied with a Gaussian kernel with an FWHM of 1 mm, using FSL.

#### Regions of interest (ROIs)

A combination of the Tohoku University Wistar Rat (Valdés-Hernández et al., 2011) and the Waxholm Space (WHS; Papp et al., 2014) atlases was used to localize the ROIs. These regions were selected for their relevance to cognitive flexibility (Leber et al., 2008; Chen et al., 2014; Dajani and Uddin, 2015; Vatansever et al., 2016). The striatum, dorsolateral orbital, frontal association, anterior cingulate (areas 1 and 2), and retrosplenial (combining the RSD, RSGb, and RSGc regions) cortices were defined as the combination of left and right portions from the Tohoku atlas, and the striatum was selected from the WHS atlas.

#### Functional connectivity analysis

Once the images were preprocessed, the average time series from each of the ROIs were extracted, Pearson’s correlation between all possible pairs was estimated, and Fisher’s z-transformation was calculated using MATLAB (MathWorks). A posterior analysis to identify sets of connections associated with age was done using network-based statistics (NBS; Zalesky et al., 2010). This method estimates the statistical significance of sets of connections by comparing their strength (the sum of their statistical weight) with that of a null distribution estimated with permutations of the original data. The sets of connections to be tested are defined as connections that show a statistical significance at the connection level (*p* < 0.05, noncorrected for multiple comparisons) and share at least one node between them. NBS naturally controls the multiple comparisons problem by defining the statistical significance at the cluster level (sets of connections) based on how probable it is to obtain such statistical strength in the null distribution, estimated with 5000 random permutations of the original data (Zalesky et al., 2010). Specifically, a one-way ANOVA was performed to identify clusters of connections with an age effect and D-serine effect. Correlation analysis was also performed between the connectivity strengths and performance measures in the cognitive tasks.

### Histology

#### Rapid Golgi neuronal staining

Fresh sections of ∼0.5 cm were cut using a blade. They were rinsed with distilled water and then immersed in a plastic container with 5-ml impregnation solution which contained mercuric chloride, potassium dichromate, and potassium chromate (solution AB) mixed 24 h in advance. Section impregnation solution was replaced 24 h after and stored at room temperature (RT) in the dark for 10 d. Sections were transferred to 6-ml solution C, which was replaced with a fresh one 24 h after and was kept for 72 h at RT in the dark. Sections were cut into 150- to 180-μm-thick slices using a sliding microtome at −80°C, collected, and mounted on gelatin-coated microscope slides. Silver nitrate (DE solution) was freshly prepared, as well as other solutions, according to the manufacturer’s instructions (FD Rapid GolgiStain kit, FD Neurotechnologies). Slides previously stained with DE solution were rinsed with Milli-Q water and then immersed in the solution for 10 min. After staining, slides were washed and dehydrated in sequential rinses of 50%, 75%, 95%, and 100% ethanol, and cleared with xylene. Slides were covered using a mounting medium (Entellan, Merck Millipore) until complete drying.

#### Morphologic quantification

Morphology analysis of the dendritic neuron projections was performed in middle-aged and aged rats from control and treatment groups. Golgi staining frontal cortex neurons were located approximately between 3.70 and 2.20 mm anterior to Bregma (Paxinos and Watson, 2006) and visualized using bright-field microscopy (Carl Zeiss Axio Imager Z3). Z-stacks were acquired with steps of 0.5 μm and a pixel size of 1 × 1 μm using a 40× objective (Plan-Apochromat 40×/1.4 Oil DIC M27, Carl Zeiss). For the dendritic feature of frontal neurons, the background was removed for each image, the seeds points were located in the soma and each dendritic branch was manually reconstructed using the filament tracer module of IMARIS software (IMARIS 9.72; Bitplane). Dendritic spines were visually identified using bright-field microscopy based on their morphologic characteristics (i.e., length, head diameter, and neck diameter; Peters and Kaiserman-Abramof, 1970). The density of spines per neuron was computed manually and double-blind on segments of 30 μm each and is expressed as the median of 5 dendritic segments. For the quantification of the thickness of proximal renal tubules, the kidneys were removed after decapitation, cut them in half and immediately immerse in formalin (10%) for fixation. The tissue was embedded in paraffin, sliced with a microtome (5 μm) and stained with hematoxylin-eosine. We use an Apotome Zeiss (Axo imager) to acquire the images (pixel size 1 × 1 μm). We randomly selected three proximal renal tubules to measure the length of the epithelial cells using the software Fiji. We computed the length of four epithelial cells per tubule located around the proximal tubule (each cell in one of the sides of the tubule) and we obtained the mean of each tubule for the purpose of the statistics. Were indicates, we perform Masson’s trichrome stain instead of hematoxylin-eosine.

### Statistics

Statistical analyses were performed using Prism (V5.01). To identify the age effect on cognitive flexibility, the thickness of proximal renal tubules and attentional task, we performed one-way ANOVA followed by Dunnet’s multiple comparison test. When two groups were compared, Student’s *t* tests were performed. Correlation analyses were also performed between the connectivity strengths and performance measures on the cognitive tasks. Significance was considered as *p *≤* *0.05

## Results

### Aging-associated cognitive flexibility decline is restored by D-serine

Cognitive flexibility is the ability to adapt behavior to a dynamically changing environment (Harada et al., 2013). To characterize age-related changes in cognitive flexibility, young (six to eight months, *n* = 36), middle-aged (18–20 months, *n *=* *49) and aged rats (24–26 months, *n *=* *33) were trained in a reversal learning task. During training sessions, the rats learned to press the lever ipsilateral to the light to obtain a reward (food pellet; Fig. 1*A*). All the groups displayed similar time courses and no significant difference was observed between groups at the end of the training sessions (Fig. 1*B*). In the reversal phase (cognitive flexibility test), the rats did not receive a reward after pressing the lever ipsilateral to the light; instead, they received it when pressing the contralateral lever (Fig. 1*C*). The persistence in responding to the previously reinforced lever (ipsilateral) 10 min after starting the session was counted as perseverative errors and considered as an inverse measurement of cognitive flexibility. Both middle-aged and aged rats had significantly more perseverative errors (∼60%) than younger rats (one-way ANOVA, *F*_(3,76)_ = 12.41, *p* < 0.0001; young vs middle-aged *p* ≤ 0.05; young vs aged *p* < 0.05, Dunnett’s test; Fig. 1*D*).

Several lines of evidence have shown that NMDAR hypofunction is a key contributor to cognitive impairments (Rowland et al., 2005; Kumar, 2015; Tanqueiro et al., 2021) including cognitive flexibility (Brigman et al., 2010; Jett et al., 2017; Baez et al., 2018; Thonnard et al., 2019; McQuail et al., 2021). In particular, an age-related decrease in D-serine levels has been reported (Potier et al., 2010). Based on this evidence, we hypothesized that the detriment in cognitive flexibility could be because of a decrease in D-serine brain levels; thus, D-serine supplementation could restore cognitive flexibility in aged animals. Given that D-serine can be absorbed in the digestive tract (Hatanaka et al., 2002), cross the blood-brain barrier (Pernot et al., 2012), and increase its levels in cortex, forebrain and hippocampus (Otte et al., 2013), we supplemented D-serine (300 mg/kg) for two months in the drinking water before evaluating cognitive flexibility.

Both middle-aged and aged rats supplemented with D-serine (300 mg/kg) had significantly fewer perseverative errors compared with control animals receiving vehicles (Fig. 1*E*, two-tailed *t* test, middle-aged vs middle-aged + D-serine, *t* = 2.03, *p* = 0.047; aged vs aged + D-serine, = 2.40, *p* = 0.022) increasing the performance in both groups. However, it was unclear whether D-serine was simply improving the performance or whether its effect was associated with aging. To address this, we analyze the effect of D-serine on young rats. In this case, young rats supplemented with D-serine did not improve their cognitive flexibility (Fig. 1*F*, two-tailed *t* test, young vs young + D-serine, *p* = 0.1421, *t* = 1.497) pointing to an age-dependent effect of D-serine.

Because D-serine supplementation can cause nephrotoxicity in young animals (Hasegawa et al., 2019), we wondered whether a lower dose of D-serine (30 mg/kg of weight) could also restore the deterioration of cognitive flexibility in aged rats. A low dose of D-serine was not sufficient to change the performance of either middle-aged or aged rats (Fig. 1*G*, two-tailed *t* test, middle-aged vs middle-aged + D-serine, *t* = 0.42, *p* = 0.67; aged vs aged + D-serine, *t* = 0.76, *p* = 0.44), supporting a dose-dependent effect of D-serine.

### D-serine partially restores functional brain connectivity decreased by aging and is relevant for cognitive flexibility performance

**Figure 2.**
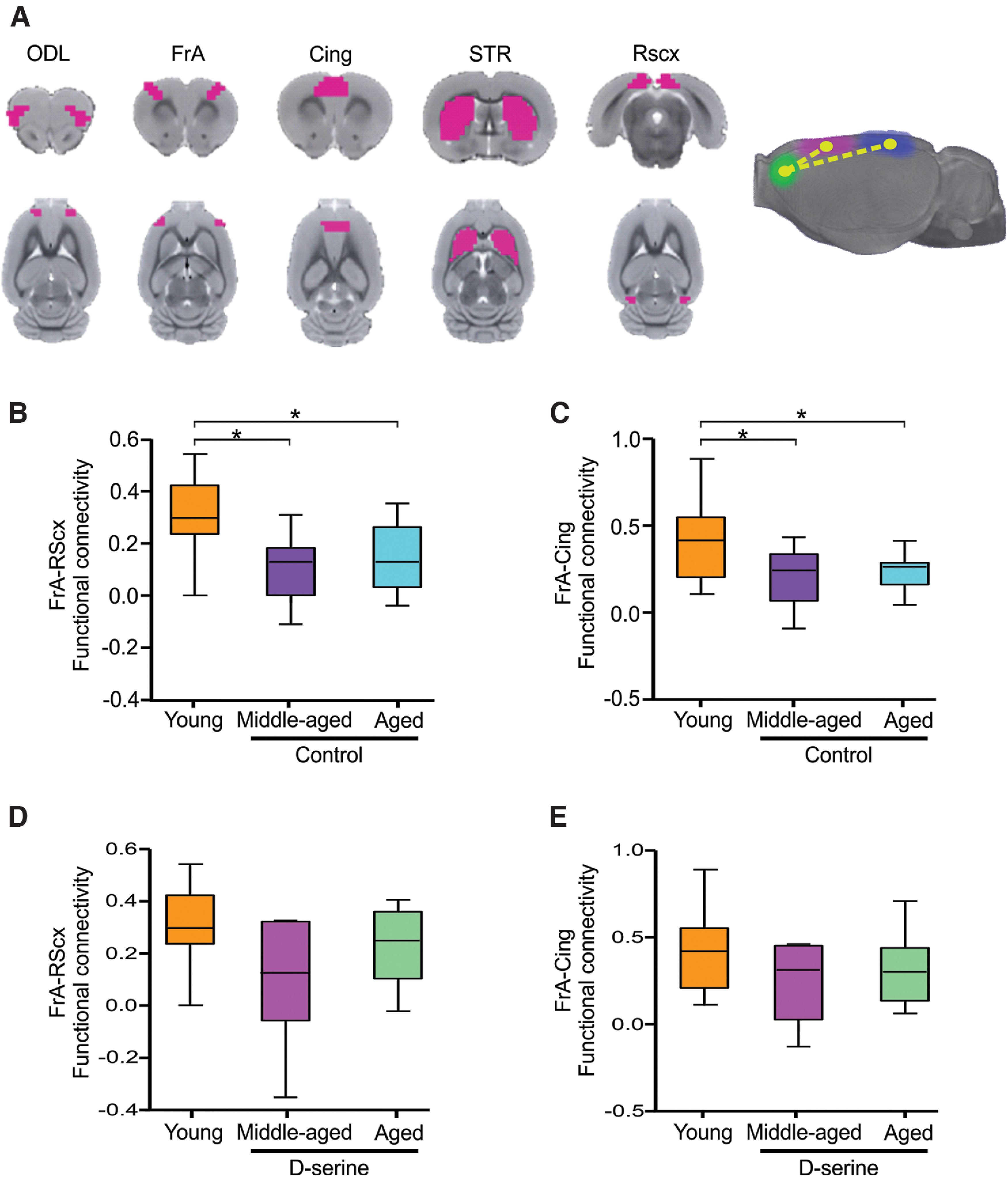
Decreased brain functional connectivity by aging is restored by D-serine. ***A***, Left, Coronal slices and axial view of the rat templates overlayed with five ROIs taken from Tohoku University and WHS atlases. Dorsolateral orbital cortex (ODL), frontal association cortex (FrA), cingulate cortex (Cing), striatum (STR), retrosplenial cortex (RScx). A brain network affected by age was identified using NBS; this network comprises FrA, Cing, and RScx cortices (right). Middle-aged (***B***) and aged rats (***C***) had less functional connectivity between FrA-RScx and FrA-Cing, respectively, compared with young rats. Middle-aged (***D***) and aged rats (***E***) that received D-serine restore the functional connectivity between FrA-RScx and FrA-Cing, respectively. Data are expressed as median ± IC 10% and 90%; *p ≤ 0.05.

Aging is characterized by functional and structural modifications that alter the brain’s functional connectivity. Because D-serine reverses the aging-associated decline in cognitive flexibility, we hypothesized that D-serine supplementation could also restore brain functional connectivity modifications because of aging. To do this, we used fMRI to characterize resting-state functional brain connectivity changes that occur during aging. For the analysis, we selected brain structures relevant for cognitive flexibility and with high expression of NMDARs (Rushworth et al., 2003; Robbins, 2007; Hyafil et al., 2009; Powell et al., 2017; Marquardt et al., 2019; Britten et al., 2020), specifically the striatum (STR), dorsolateral orbital (ODL), frontal association (FrA), anterior cingulate (Cing), and retrosplenial (RScx) cortices (Fig. 2*A*, left).

**Figure 3.**
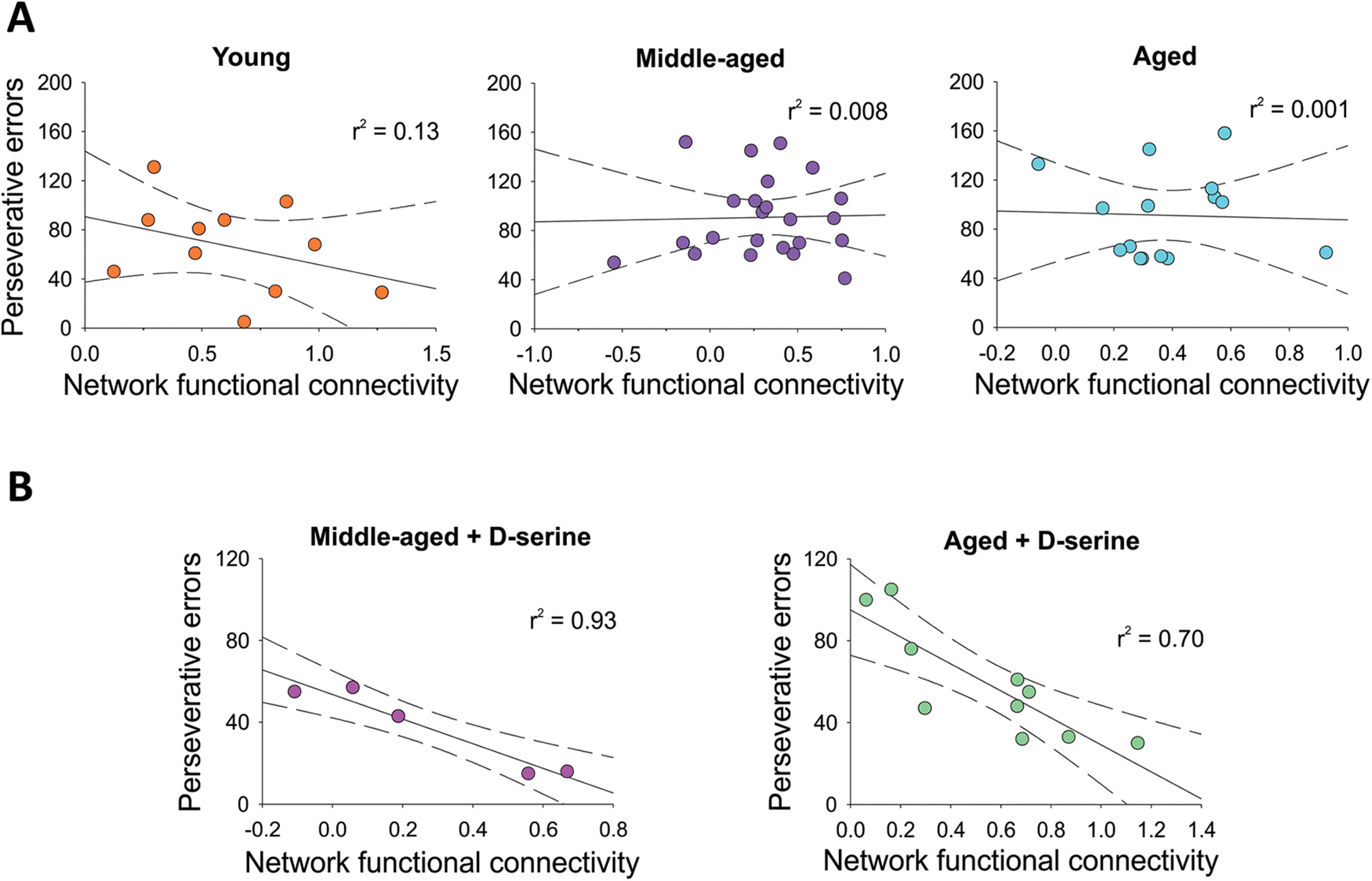
D-serine makes functional brain connectivity relevant for cognitive flexibility performance. ***A***, Young, middle-aged and aged rats receiving vehicle did not show a correlation between the network functional connectivity and the perseverative errors. ***B***, Chronic D-serine supplementation to middle-aged and aged rats had a negative correlation between the network functional connectivity and the perseverative errors. Middle age + D-serine, *r*^2^ = 0.93 *p* = 0.0068; aged + D-serine, *r*^2^ = 0.070 *p* = 0.0023.

Using the NBS Toolbox (Zalesky et al., 2010), we identified a brain network that is affected by aging composed of three nodes: frontal association, retrosplenial and cingulate cortices, and two functional connections between them (FrA-RScx and FrA-Cing; Fig. 2*A*, right). A posteriori tests allowed us to identify the behavior of the individual connections: middle-aged and aged rats showed a significant decrease in the functional connectivity between frontal association and retrosplenial cortices (Fig. 2*B*, FrA-RScx: one-way ANOVA, *F*_(3,51)_ = 7.09, *p* = 0.0019; young vs middle-aged *p* < 0.05; young vs aged *p* < 0.05 Dunnett’s test), as well as a decrease in the connectivity between frontal association and cingulate cortices (Fig. 2*C*, FrA-Cing: one-way ANOVA, *F*_(3,51)_ = 6.32, *p* = 0.0035; young vs middle-aged *p* < 0.05 young vs aged *p* <0.05 Dunnett’s test). We then tested whether D-serine was effective in restoring the functional brain network decreased by aging. We did not observe significant changes in middle-aged and aged rats supplemented with D-serine compared with those receiving vehicles (FrA-RsCx: middle-aged vs middle-aged + D-serine, *p* = 0.9534; *t* = 0.0588; FrA-RsCx: aged vs aged + D-serine, *p* = 0.1771, *t* = 1.387; FrA-Cing: middle-aged vs middle-aged + D-serine, *p* = 0.7197, *t* = 0.3623; FrA-Cing: aged vs aged D + serine; *p* = 0.2204, *t* = 1.256). However, the functional connectivity between frontal association with retrosplenial (Fig. 2*D*) and cingulate cortices (Fig. 2*E*) were also not statistically different compared with young rats showing that D-serine partially preserves the functional connectivity that is affected by aging (FrA-RScx: one-way ANOVA, *F*_(3,31)_ = 2.65, *p* = 0.086; FrA-Cing one-way ANOVA, *F*_(3,31)_ = 1.76, *p* = 0.18). We then analyzed whether the increase in brain functional connectivity between frontal association cortex and cingulate and retrosplenial cortices could be associated with the restoration of cognitive flexibility in senescent animals supplemented with D-serine. The performance of young, middle-aged and aged rats in the reversal learning task (perseverative errors) was not correlated with their brain network connectivity (young: *r*^2^ = 0.13. *p* = 0.27, middle-aged: *r*^2^ = 0.0008 *p* = 0.89; aged: *r*^2^ = 0.0015 *p* = 0.88; Fig. 3*A*), meaning that the increase in perseverative errors is not exclusively because of a decrease in the connectivity of this network. However, rats chronically supplemented with D-serine showed a negative correlation between the number of perseverative errors and the strength of the functional connectivity between the frontal cortex and cingulate and retrosplenial cortices (middle-aged + D-serine *r*^2^ = 0.93 *p* = 0.0068; aged + D-serine, *r*^2^ = 0.070 *p* = 0.0023; Fig. 3*B*). These results reveal that D-serine reversed the decline in cognitive flexibility in senescent rats by increasing the functional connectivity within this brain network pointing to the frontal association cortex as the hub of D-serine effects regulating prefrontal cortex-dependent executive function associated with senescence.

### D-serine increases frontal neuron spines in middle-aged and aged rats

**Figure 4.**
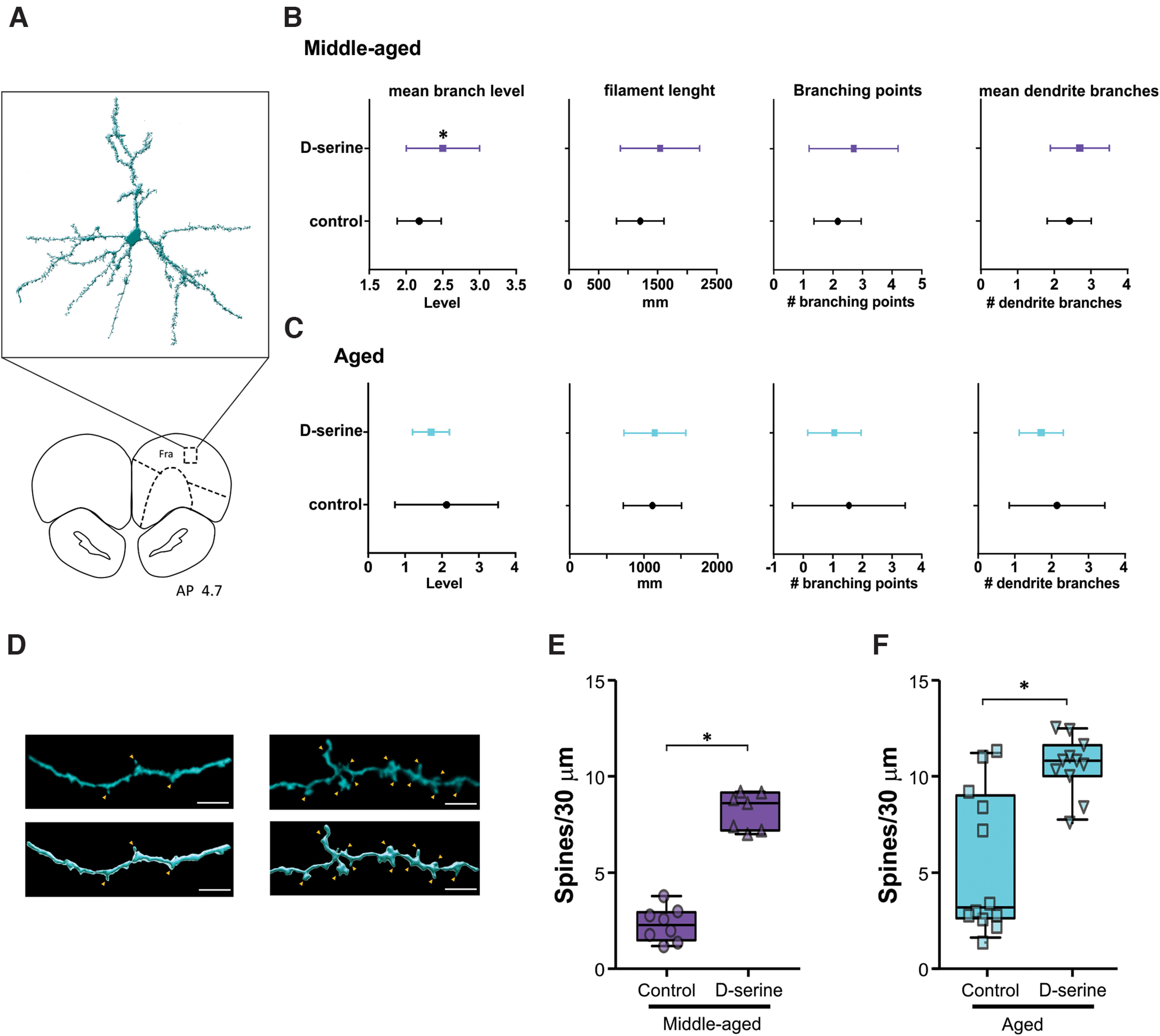
D-serine increases frontal neuron spines without affecting dendritic features. ***A***, 3D reconstruction of a typical frontal neuron of an aged rat supplemented with D-serine. Morphologic features of frontal neuron dendrites of middle-aged (***B***) and aged rats (***C***) receiving vehicle or D-serine. ***D***, Representative image and 3D reconstruction of dendritic segments of middle-aged and aged rats receiving vehicle (left) and supplemented with D-serine (right). Orange arrowheads indicate spines. Scale bar: 5 μm. Population density of frontal spines of middle-aged (***E***) and aged rats (***F***) in control conditions and supplemented with D-serine; two-tailed *t* test, *p ≤ 0.05.

Aging-related decline in cortical functional connectivity has been associated with changes in morphologic neuronal features, such as a decrease in the dendritic branching and a reduction of neuronal spines (Feldman and Dowd, 1975; Mostany et al., 2013). Because D-serine regulates neuronal dendritic arborization and spine density in young and adult animals (Balu and Coyle, 2014; Zou et al., 2016), we wonder whether these could be the cellular mechanisms underlying D-serine effects on frontal functional connectivity with cingulate and retrosplenial cortices. To assess this, we performed 3D reconstructions of Golgi-stained frontal neurons (Fig. 4*A*) and quantified morphologic features such as mean branch level, filament length, branching points, and dendritic branches. Middle-aged but not aged rats receiving D-serine exhibited a significant increase in the mean branch level compared with controls (Fig. 4*B*, two-tailed *t* test, middle-aged vs middle-aged + D-serine, *t* = 0.076, *p* = 0.032) without any significant changes in the other parameters (Fig. 4*C*). We then quantified the density of frontal dendritic spines, resulting in a significant increase in the number of total spines in middle-aged and aged rats supplemented with D-serine compared with those receiving only vehicle (Fig. 4*E*, middle-aged vs middle-aged + D-serine, *t* test = 12.35, *p* < 0.0001; aged vs aged + D-serine, *t* test = 4.26, *p* = 0.0003).

**Figure 5.**
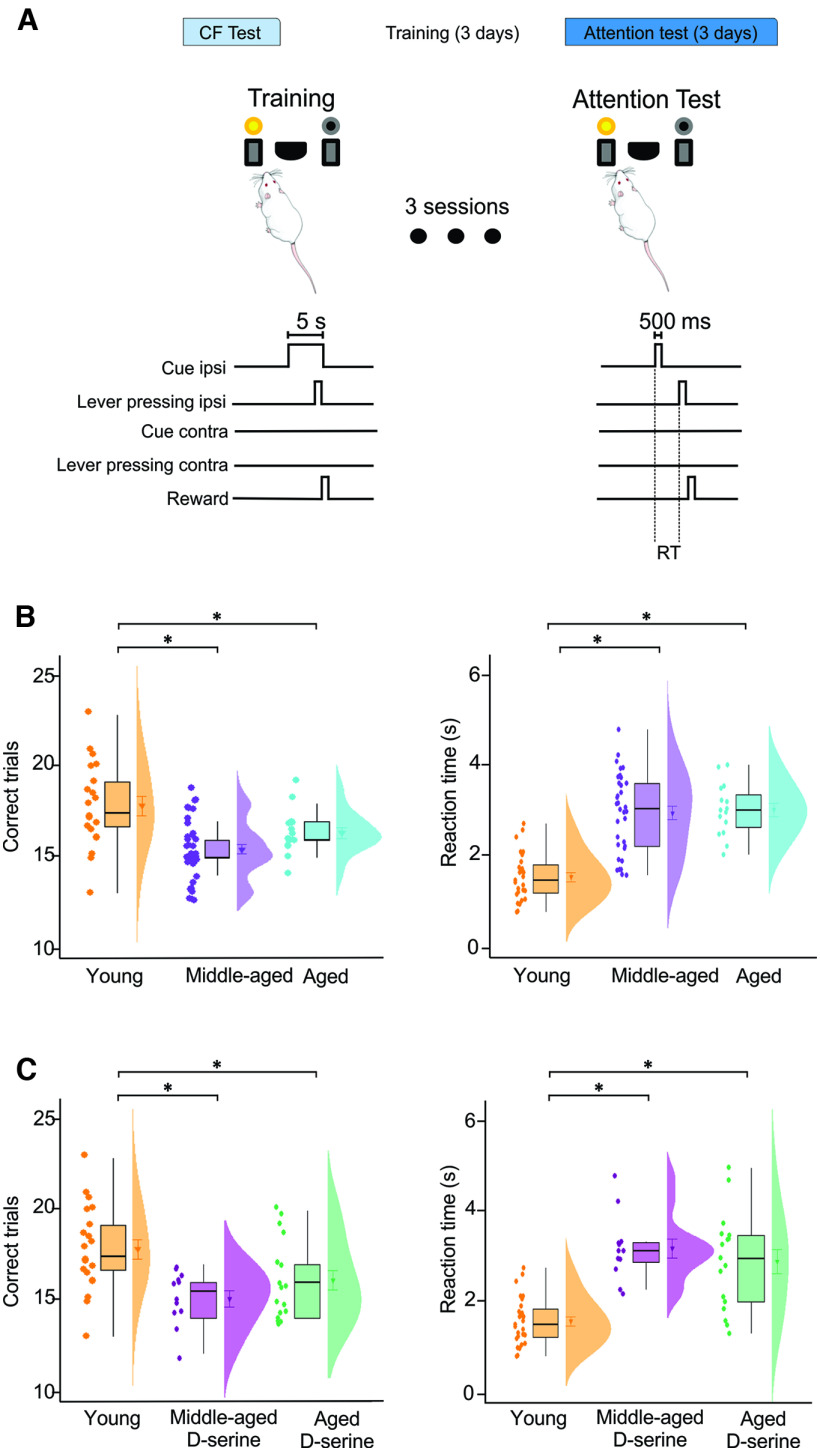
D-serine did not affect attentional components decreased with age. ***A***, Behavioral task design during training and attention test sessions. A correct trial was counted when the rat pressed the level ipsilateral to the light. Reaction time was determined as the time occurring between the light was switched off and the ipsilateral lever was pressed. ***B***, The number of correct trials significantly decreased (left) and reaction time significant increased (right) in middle-aged and aged rats compared with young rats. ***C***, Correct trials (left) and reaction time (right) were not modified by D-serine supplementation in middle-aged and aged rats. Data are expressed as median ± IC 10% and 90%. One-way ANOVA; * *p* ≤ 0.05.

To examine whether D-serine effects could extend to other domains of brain function, such as attentional components that could also be involved in cognitive flexibility, young, middle-aged, and aged rats were retrained to press the lever ipsilateral to the light (correct trial) until reaching 70% of correct trials. As a measurement of the attentional component, the day of the test we decreased the duration of the light (0.5 s) and quantified the time the animals took to respond (reaction time), as well as the number of correct choices (pressing the correct lever; Fig. 5*A*). Using this task, we observed a decrease of both parameters in the senescent groups compared with young rats (Fig. 5*B*), showing a detriment in the attentional processes because of aging (Fig. 5*B*, correct trials, one-way ANOVA, *F*_(3,68)_ = 11.49, *p* < 0.0001; young vs middle-aged *p* < 0.05; young vs aged *p* < 0.05, Dunnett’s test. Reaction time, one-way ANOVA, *F*_(3,69)_ = 6.22, *p* = 0.0033; young vs middle-aged *p* < 0.05; young vs aged *p* < 0.05, Dunnett’s test). We then tested whether D-serine supplementation was also able to revert this detriment (Fig. 5*C*, correct trials, one-way ANOVA, *F*_(3,46)_ = 7.008, *p* = 0.0022; young vs middle-aged *p* < 0.05; young vs aged *p* < 0.05, Dunnett’s test. Reaction time, one-way ANOVA, *F*_(3,48)_ = 22.16, *p* < 0.0001). However, in this case, D-serine supplementation was not able to restore the detriment of attention in aged rats, suggesting that D-serine is not a general cognitive enhancer for aged subjects.

### D-serine does not cause nephrotoxic damage in middle-aged or aged rats

**Figure 6.**
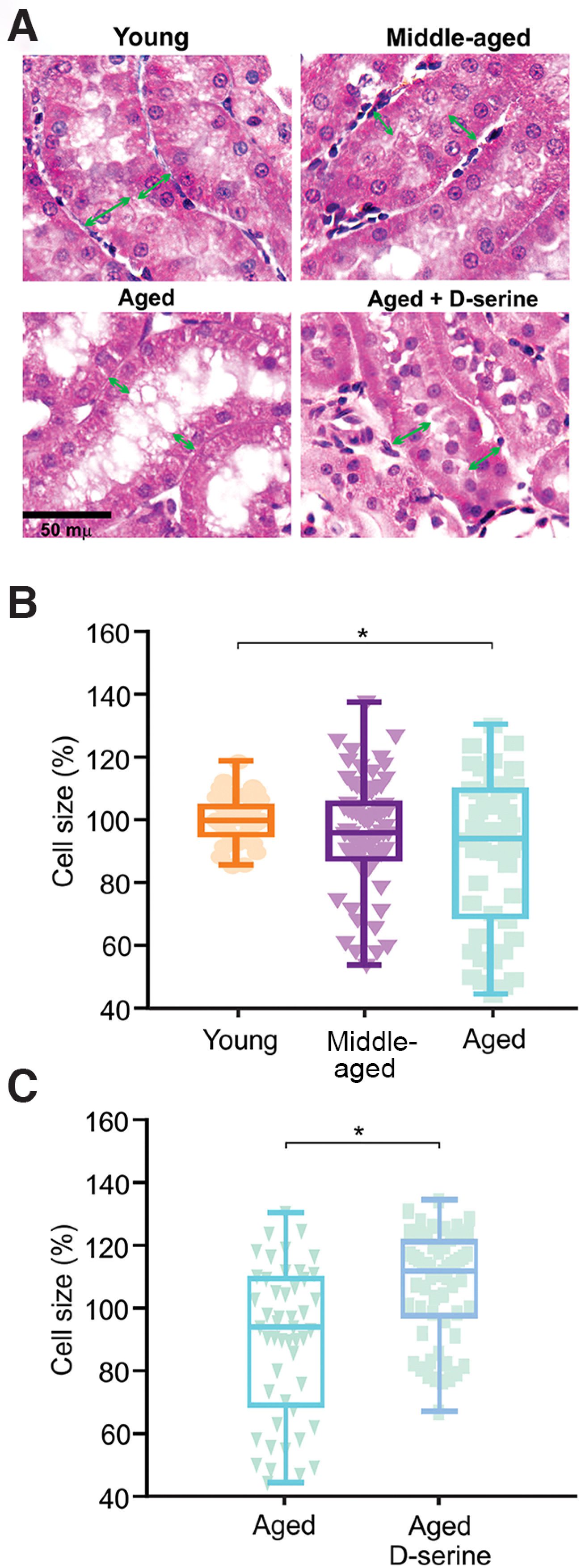
D-serine increased the cell size of proximal tubules in the kidney. ***A***, Representative images of kidney proximal straight tubules from young, middle-aged, aged, and aged rats supplemented with D-serine. The tissue is stained with Masson’s trichrome. Orange arrows indicate the diameter of the cells analyzed. ***B***, Middle-aged and aged rats show a significant decrease in the size of tubular cells compared with young rats. Cell size was normalized in relation to the mean of young cells. ***C***, D-serine increased the diameter of tubular cells in the proximal straight tubule. Data are expressed as mean + maximum and minimum values. Two-tailed *t* test; *p ≤ 0.05. Scale bar: 50 μm.

D-serine supplementation in senescent animals restores the aging-associated decline in cognitive flexibility, functional connectivity, and spine density. However, D-serine is catabolized in the straight proximal tubule of the nephron producing oxide peroxide, which could damage the kidney cells. Although the dose of D-serine supplemented to our rats has been reported as safe for young animals (Hasegawa et al., 2019), we were concerned about possible nephrotoxic damage in our aged animals (Hasegawa et al., 2019). To test this, we used Masson’s trichrome stain to evaluate the integrity of the proximal straight tubule by means of fibrin staining from collagen (Fig. 6*A*). Aged rats supplemented with D-serine showed a decrease in damaged renal tubules based on the double-blind quantification of Masson’s trichrome stain (57% vehicle vs 20% D-serine), indicating that D-serine does not affect the tissue integrity of the straight proximal renal tubules. However, as a normal process of aging, there is a detriment in the function of proximal straight tubules, which is histologically manifested as tubular atrophy, dilation, interstitial fibrosis and a reduction of the tubular microvellosities and the thickness of endothelial cells (Nakano et al., 1985). To strengthen our histologic analysis, we computed the diameter of the endothelial cells of young, middle-aged and aged rats receiving vehicle or D-serine. Our results show a decrease in the thickness of endothelial tubular cells in aged rats compared with young rats (Fig. 6*B*, one-way ANOVA, *F*_(3,183)_ = 5.16, *p* = 0.006; young vs middle-aged *p* > 0.05; young vs aged *p* < 0.05, Dunnett’s test) However, D-serine supplementation restores the diameter of the endothelial cells, making it comparable to that of younger rats (Fig. 6*C*, two-tailed *t* test, aged vs aged + D-serine, *t* = 0.462, *p* < 0.0001).

## Discussion

Pharmacological interventions in the aging field aim to retard, prevent, decrease, or reverse age-related brain alterations. Here, we show that chronic supplementation of D-serine to senescent rats reverts the decrease in cognitive flexibility, functional brain connectivity, and frontal neuronal spine density that is affected in aged animals. We found that D-serine supplementation decreases the number of perseverative errors in a reversal learning task in middle-aged and aged rats by increasing the functional connectivity between frontal association areas with retrosplenial and cingulate cortex. Furthermore, D-serine supplementation did not induce nephrotoxicity; instead, it restored the thickness of the epithelial tissue in the straight portion of proximal renal tubules of senescent rats suggesting that D-serine can reverse the detriment of aging-associated malfunction of peripheral tissue (Rivera-Villaseñor et al., 2021). D-serine did not improve cognitive flexibility in young rats showing that D-serine effect is age-dependent pointing to a possible intervention in restoring the levels of D-serine to reverse cognitive functions that are affected in the aged brain.

Cognitive flexibility is the ability to adapt the behavior to a changing environment (Harada et al., 2013), switching between sets of responses to generate new strategies to solve problems (Scarmeas et al., 2003). Failures in this brain function are associated with persistent behavior in which an individual continues to follow the same rule although they are failing the task. Cognitive flexibility starts to decrease at the beginning of middle age in humans (∼40 years of age) and rats (∼12 months of age; Reimers and Maylor, 2005; Beuk et al., 2016), which is consistent with the detriment observed in our old rats. Although the precise mechanisms responsible for the aging decline in cognitive flexibility are unclear, NMDAR plays a pivotal role. Thus, NMDAR blockade induces deficits in reversal learning tasks, increasing the perseverative behavior in mice (Thonnard et al., 2019) and young rats (van der Meulen et al., 2003). Furthermore, cognitive flexibility impairments that involve NMDAR hypofunction are commonly observed in patients with schizophrenia (Wobrock et al., 2009). Here, we showed that the NMDAR co-agonist D-serine, orally supplemented for two months in the drinking water, fully restored cognitive flexibility in middle-aged and aged rats. This raises the possibility that our D-serine supplementation could restore brain D-serine levels affected by age that could improve NMDAR function. However, additional experiments analyzing the effect of D-serine of NMDAR activity would be required to clarify this.

Previous works have identified brain regions that are active when a person engages in cognitive flexibility tasks, including the prefrontal cortex, basal ganglia, hippocampus, and cingulate cortex (Leber et al., 2008; Chen et al., 2014; Dajani and Uddin, 2015; Vatansever et al., 2016). These brain structures are also related to cognitive flexibility in rodents (Brockett et al., 2015; Anacker and Hen, 2017), suggesting homologous brain network organization related to this cognitive function among species.

During the normal aging process, functional brain connectivity is altered (Andrews-Hanna et al., 2007; Varangis et al., 2019; Cao et al., 2021), particularly in regions comprising the Default Mode Network, which mediates executive functions (J.T. Wu et al., 2011; Chou et al., 2013; Madden et al., 2020). These regions include frontal areas, cingulate cortex, retrosplenial cortex, and hippocampus (Hafkemeijer et al., 2012; Salami et al., 2014; Oren et al., 2019), as well as sensory and motor areas (Kiyama et al., 2014; Wang et al., 2019). Here, we identified an aging brain network in rats comprising three nodes (frontal association areas, cingulate and retrosplenial cortices) and two connections (frontal-cingulate cortex and frontal-retrosplenial cortex) that displayed a marked reduction in the resting-state functional connectivity in middle-aged and aged subjects compared with young rats. In concordance, the integrity of a large-scale network involving medial frontal, retrosplenial cortex, posterior cingulate cortex, and medial temporal regions becomes less correlated in elder subjects (Andrews-Hanna et al., 2007; Ziontz et al., 2021), reinforcing homologous systems and mechanisms in the aging process and making rats a good model to study large-scale brain dynamics and its relation to cognitive functions (Zhao et al., 2008; Ferrari et al., 2012; Lu et al., 2012). In the present work, we aimed to analyze D-serine effects on aging-related alterations in large-scale brain systems that could support cognitive flexibility. Chronic supplementation of D-serine fully restored the aging-associated reductions in the functional connectivity of this aging network, in concordance with the high expression of NMDAR and the location of D-serine in frontal areas and the cingulate and retrosplenial cortices (Schell et al., 1997). Although the strength of these functional connectivities in the resting state does not correlate with the perseverative errors in control rats, the animals supplemented with D-serine showed a positive relationship between the functional connectivity of frontal areas with cingulate and retrosplenial cortices and their performance in the flexibility task. This suggests that D-serine may compensate for aging-associated deficits by reorganizing large-scale networks to use brain areas not used in control subjects to improve the performance of old rats.

Although the precise substrate underlying functional brain connectivity measured with BOLD-signal is unclear, it is related to brain features (Mueller et al., 2018) such as cortical thickness (Salat et al., 2004; Thambisetty et al., 2010), the complexity of dendrite ramifications, and the density of dendritic spines (Smith, 2002; Marcar and Loenneker, 2004). Dendrite spines are dynamic structures that undergo remodeling modifying synaptic strength and neuronal plasticity. High levels of D-serine during development correlate with periods of dynamic plasticity and synaptogenesis (Hashimoto et al., 1993; Fuchs et al., 2006). In young adults, D-serine levels decrease but they are still sufficient to maintain and promote spinogenesis (Balu et al., 2012; Sultan et al., 2013) through NMDA-dependent mechanisms (Panatier et al., 2006; Perez-Rando et al., 2017) and restore deficits in spine dynamics, morphology and neuronal plasticity in amyloid precursor protein knock-out mice (APP-KO). In agreement with this, we show that D-serine chronically supplemented to senescent rats increases frontal neuronal dendrites in middle-aged and aged rats which can underlie the D-serine effects on functional connectivity and cognitive flexibility.

D-serine brain levels depend on the balance between its synthesis from serine racemase (SR), the enzyme responsible for racemization of L-serine to D-serine, and its catabolism from D-amino acid oxidase (DAAO) in the brain. Also, D-serine can leave the brain by crossing the blood-brain barrier (through ATB0 transporters) to be degraded in the renal proximal straight tubule where DAAO is abundant. There is currently a debate about the source of D-serine in the brain. While some authors have shown that D-serine and SR are mainly localized in astrocytes (Schell et al., 1995; Papouin et al., 2012; Koh et al., 2022), others have proved they are present exclusively in neurons (Miya et al., 2008; Balu and Coyle, 2014; Wolosker et al., 2016). Whether brain D-serine is derived from neurons or astrocytes, D-serine content is decreased in aged subjects (Billard, 2015; Ploux et al., 2021). This has been attributed to a reduction of SR expression because DAAO levels do not change during aging (Potier et al., 2010). However, there is no information regarding the effect of D-serine transporters in the blood-brain barrier during aging that could be involved in the reduction of brain D-serine. Our findings showed that oral supplementation of D-serine restores aging-associated deficits at the cellular and functional levels. This supports that D-serine transporters in the intestine (ASCT1, ASCT2), as well as ATB0 in the brain of senescent rats, are functional (Foster et al., 2016; Kaplan et al., 2018). However, further work will be needed to clarify how D-serine transporters are affected during aging. It will also be interesting to know whether the difference in D-serine brain levels between subjects and the variability of the effect of D-serine supplementation in aged subjects depends on the functioning of these receptors. Our results raise the possibility that restoring the brain levels of D-serine by oral supplementation at low doses of this amino acid could potentially be used as a therapeutic target to recover brain alterations associated with aging, brain functional connectivity, and behavioral performance without inducing nephrotoxicity.

## Synthesis

Reviewing Editor: Alexey Semyanov, Shemyakin-Ovchinnikov Institute of Bioorganic Chemistry Decisions are customarily a result of the Reviewing Editor and the peer reviewers coming together and discussing their recommendations until a consensus is reached. When revisions are invited, a fact-based synthesis statement explaining their decision and outlining what is needed to prepare a revision will be listed below. The following reviewer(s) agreed to reveal their identity: NONE. Note: If this manuscript was transferred from JNeurosci and a decision was made to accept the manuscript without peer review, a brief statement to this effect will instead be what is listed below.

I have read the manuscript and authors’ reply to reviewers’ comments in the Journal of Neuroscience. I agree with the reviewers that the manuscript would benefit from more mechanistic insight related to measuring the function of NMDA receptors. Since the authors cannot do such experiments, they cannot claim their data support ’that NMDAR hypofunction is a major contributor to age-associated morphological and functional changes’. One solution in this situation would be to remove such a claim from the abstract and downplay it in the text.
